# Analyzing the Application of the Sustainable Development Goals for Egypt Using a Neutrosophic Goal Programming Approach

**DOI:** 10.1007/s41660-022-00265-z

**Published:** 2022-09-03

**Authors:** Elsayed Badr, Essam El Seidy, Amani Elrayes, Aya Rabie

**Affiliations:** 1 Faculty of Computers and Informatics, Benha University, Banha, Egypt; 2 Mathematics Department, Faculty of Science, Ain Shams University, Cairo, Egypt; 3 Planning Techniques Center, Institute of National Planning, Cairo, Egypt

**Keywords:** Sustainable Development Goals, Multi-criteria decision making, Fuzzy goal programming, Neutrosophic goal programming

## Abstract

Sustainable development necessitates the implementation of appropriate policies that integrate multiple competing objectives on economic, environmental, energy, and social criteria. Multi-criteria decision analysis with goal programming is a popular and widely used technique for studying decision problems with multiple competing objectives. Real-world situations frequently involve imprecise and incomplete information, making neutrosophic goal programming models the most appealing option. We presented a novel neutrosophic goal programming model that incorporates optimal resource allocation to simultaneously satisfy prospective goals on economic development, energy consumption, workforce, and greenhouse gas emission reduction by 2030, as applied to Egypt’s key economic sectors in this paper. We also compared the outcomes of fuzzy goal programming and neutrosophic goal programming. We show that neutrosophic goal programming approach is more accurate than fuzzy goal programming approach because it deals with incomplete and indeterminate information and has three independent degrees: truth membership degree, indeterminacy–membership degree, and falsity–membership degree. The presented model examines opportunities for improvement and the effort required to implement sustainable development plans. The model also provides valuable insights to decision makers for strategic planning as well as investment allocations for sustainable development. Numerical illustration is also provided for validation and application of the proposed model.

## Introduction

Sustainable development is a philosophy that aims to achieve human development goals while also protecting natural systems’ ability to supply the natural resources and ecosystem services that the economy and society rely on. The targeted outcome is a state of society in which living circumstances and resources are utilized to suit human needs without jeopardizing the natural system’s integrity and stability (Sustainable Development 2015). Sustainable development is defined as the process of maintaining production by substituting resources of equal or better value for those used without hurting or endangering normal biological systems (Kahle and Gurel–Atay 2014).

Sustainable Development Goals (SDGs) are a series of 17 interconnected global goals aimed at making the world a better and more sustainable place for everyone (United Nations 2017). The United Nations General Assembly established the SDGs in 2015, with the aim of achieving them by 2030 (United Nations 2015). The Post-2015 Development Agenda includes the SDGs. 2015 as the starting point for a new global development framework to replace the Millennium Development Goals, which expired in 2015.

Egypt is the most populous Arab country, the third most populous African country after Nigeria and Ethiopia, and the fourteenth most populous country in the world. The population of Egypt in 2021 was about 102 million. Egypt’s population is predicted to reach 120 million by 2030.^1^ It is noted that Egypt’s population growth rate is significantly high, which has a significant impact on the state’s development efforts and necessitates more effort from all parties to limit population growth and reduce this rate to achieve balance with economic growth.

Despite the negative effects of the “Covid-19” pandemic on global and Arab economies, Egypt was able to mitigate the pandemic’s effects; this was due to an economic reform program implemented since 2016. During the first phase of the economic reform program, the Egyptian economy achieved several successes, including an increase of gross domestic product (GDP), a reduction in the budget deficit. Egypt is anticipated to rank third among the top Arab economies in 2021, with GDP of $394.3 billion.^2^

Energy is the main pillar and lifeline for development in various fields of economic and social life, with the sector emphasizing its ongoing efforts to provide electrical energy to all of its applicants on time and with sufficient capacity, as well as to expand the use of new and renewable energies. Energy consumption is deemed sustainable if it serves current demands without jeopardizing future generations’ needs. In the near term, Egypt was able to meet its plan, which it had intended to attain in 2022, by achieving a contribution of renewable energy capacity of 20% of total electricity produced in 2020. Egypt’s renewable energy production increased by around 2000 MW in 2019, with hydropower accounting the most of the growth with about 2835 MW, solar power with about 1740 MW, and wind power with about 1375 MW. Due to the use of petroleum fuels in power plants, Egypt’s energy sector is the leading source of carbon dioxide emissions ${(\mathrm{CO}}_{2})$. ${\mathrm{CO}}_{2}$ emissions have been lowered as a result of improved fuel efficiency in power plants and increased usage of renewable energy.^3^

Egypt’s 2030 unemployment strategy intends to encourage small and micro businesses, resulting in reducing both unemployment rate and poverty rate. The data of the Central Agency for Public Mobilization and Statistics showed that the poverty rate in Egypt fell to 29.7% in 2019/2020, for the first time in 20 years. Also, the income and expenditure research indicators for the year 2019/2020 showed a remarkable decline in the poverty rate to reach 29.7%, compared to 32.5% in 2017/2018.^4^,^5^ According to a report by the Media Center of the Egyptian Cabinet, the unemployment rate declined by 5.2 percentage points, to record 7.3% in the second quarter of 2021, compared to 12.5% in the second quarter of 2016. Despite the Corona pandemic, Egypt aims to reach 7.9% until the year 2025.^6^

In 1965, Zadeh introduced fuzzy set theory (Zadeh 1965), which is an applied approach to overcoming uncertainty by assigning a membership function to any non-deterministic event. Atanassov introduced the intuitionistic fuzzy sets, which are an extension of fuzzy sets, in 1986 (Atanassov 1986). Intuitionistic fuzzy sets consider both the truth membership function ${T}_{\tilde{A }}\left(x\right)$ and the falsity membership function ${F}_{\tilde{A }}\left(x\right)$. Intuitionistic fuzzy sets are incapable of dealing with inconsistent and indeterminate information, both of which are common in belief systems.

We frequently encounter incomplete and indeterminate information in real-world scenarios, so the information cannot be represented merely by the membership function and non-membership function. In 1995 (Smarandache 1995), Smarandache proposed the neutrosophic set which deals with incomplete and indeterminate information and is distinguished by three independent degrees: truth membership degree, indeterminacy-membership degree, and falsity-membership degree.

Han et al. (Han et al. 2012) proposed a multi-objective optimization model for determining viable technologies for producing electricity and treating ${\mathrm{CO}}_{2}$ with the goal of maximizing predicted revenues while minimizing financial risk. Flores et al. (Flores et al. 2014) proposed a mathematical programming methodology for energy investment planning to maximize the Net Present Value over time. The model incorporates renewable and non-renewable demands, new energy facility sources, and the present amount of fossil-fuel reserves. Gupta et al. (Gupta et al. 2018) created a fuzzy goal programming model that allocates resources optimally by accomplishing future goals in terms of gross domestic product, electricity consumption, and greenhouse gas emissions. Chang (2015) used a goal programming approach to identify the key ${\mathrm{CO}}_{2}$ generating industries to optimize production structure in order to meet China’s emission reduction targets.

Schult et al. (2014) proposed mixed integer linear programming methods for solving large-scale input–output systems that represent an optimal allocation of the world’s resources for a more sustainable global economy. Pal and Kumar (Pal and Kumar 2013) show how to tackle thermal power generation and dispatch problems with interval data uncertainty using a linear goal programming (GP) technique. Balaman and Selim (2014a, b) use multiple fuzzy goal programming (FGP) methodologies to solve a multi-objective optimization problem of biomass to energy supply chains in an uncertain environment. In the United Arab Emirates, Jayaraman et al. (2015, 2017) created a mathematical model that incorporates optimal resource allocation to simultaneously achieve anticipated goals on economic development, energy consumption, workforce, and greenhouse gas (GHG) emission reduction.

Abdel-Basset et al. (2018) used neutrosophic set for decision making and evaluation method to evaluate and determine the factors influencing the selection of supply chain management suppliers. Ahmad et al. (2019) provided a multi-objective optimization framework for an overall water management system that combines freshwater allocation for hydraulic fracturing and optimal wastewater control using several methodologies. Islam et al. (Islam and Deb 2019) developed a supplier selection problem with the objectives of minimizing net cost, minimizing net rejections, minimizing net late deliveries, and minimizing net GHG, all while considering realistic restrictions such as supplier capacity and buyer demand. The buyer’s desire is fuzzy in nature due to uncertainty, and it can be represented as a triangular neutrosophic number.

Hezam et al. (2016) introduced the goal programming in neutrosophic environment. The degrees of acceptance, indeterminacy, and rejection of objectives are simultaneously considered. They suggested two models for solving the neutrosophic goal programming problem (NGPP), one seeking to minimize the total of the deviation (the 1st model), and the other changing NGPP into a crisp programming model by employing truth, indeterminacy, and falsity membership functions (the 2nd model).

In this paper; we present a novel neutrosophic goal programming model that includes an optimal allocation of resources to simultaneously meet potential targets for economic development, energy consumption, labor force, and greenhouse gas emissions reduction by 2030 with application to key economic sectors in Egypt. We also compared the results of fuzzy goal programming and neutrosophic goal programming to achieve the best outcomes for the decision maker.

## Multi-Criteria Fuzzy Goal Programming Formulation

Goal programming is a popular multi-criteria decision-making technique that is based on the distance function concept, in which the decision maker (DM) seeks the solution that minimizes the absolute deviation between the objective’s achievement level and its aspiration level. It was proposed by (Charnes et al. 1955) in a model for executive compensation. At the time, the term “goal programming” was not used, and the model was viewed as an adaptation of linear programming.

GP is a linear programming extension that can handle multiple objectives. It is used to solve a multi-objective optimization problem that balances a trade-off in conflicting objectives. It is also used for three types of analysis:

- Determine the resources needed to achieve a desired set of goals.
- Determine the degree of goal attainment with the available resources.
- Provide the most satisfying solution with varying amounts of resources and goal priorities.

### Mathematical Formula for Goal Programming

There are two ways to look at goal programming. In the first instance, it is an extension of linear programming to include multiple objectives, which are expressed through the attempted achievement of goal values. In the second instance, linear programming is a subset of goal programming with a single goal. These two considerations indicate that goal programming is part of the multi-objective programming paradigm (Jones and Tamiz 2003).

Say ${Z}_{i}\left(X\right)$, *X* = (${x}_{1}, {x}_{2}, \dots , {x}_{n}$), and these acceptable aspiration levels ${ g}_{i}$ (*i* = 1, 2, …, *K*). Therefore, GP can be expressed as follows:

1 $$\mathrm{Minimize}\sum\nolimits_{i=1}^k\left|Z_i\left(X\right)-g_i\right|\mathrm{Subject}\;\mathrm{to}:\;\;\;\;\;\;\;\;\;\;\;\;\;\;\;\;\;\;\mathit\;x\;\mathit\in\mathit\;X\mathit=\mathit\{x\mathit\in R^n\mathit;A_x\mathit\leq b\mathit;x\mathit\geq\mathit0\mathit\}$$

where ${Z}_{i}$ is the linear function of the $i$ th goal and ${g}_{i}$ is the aspiration level of $i$ th goal.

$${Z}_{i}\left(X\right)-{g}_{i}= {d}_{i}^{+}-{d}_{i}^{-}; {d}_{i}^{+}, {d}_{i}^{-}\ge 0$$

Here, $K$ is the total number of goals, $b$ is the right-hand side of the constraint coefficient, ${Z}_{i}\left(x\right)$ is the *k*th objective, and ${g}_{i}$ is the aspiration level of the *k*th goal. Equation (1) can be formulated as follows:

2 $$\begin{array}{l}\mathrm{Minimize}\sum\nolimits_{i=1}^k\left|d_i^+-d_i^-\right|\\\mathrm{Subject}\;\mathrm{to}:\\\begin{array}{cc}&\begin{array}{l}\;F_i\left(x\right)-d_i^++d_i^--g_i=0;i=1,2,\dots,k\\x\;\in X=\{x\in R^n;A_x\leq b;x\geq0\}\end{array}\end{array}\end{array}$$

where ${d}_{i}^{+}$ and ${d}_{i}^{-}\ge 0$ are, respectively, under and over deviations of $i$ th goal.

Any optimization model that reflects real-world scenarios contains a large number of parameters whose values are assigned based on expert opinion, and they are necessary to specify an exact value for the parameters in the traditional manner. However, both specialists and decision makers frequently do not have a precise understanding of the value of those criteria. If exact values are suggested, they are merely statistical inferences based on previous data, and their stability is questionable; hence, the problem’s parameters are typically determined by the decision maker in an uncertain area. As a result, it is beneficial to consider expert opinion about the parameters as fuzzy data, which assists the decision maker in making a decision in an open-ended space; because the market is volatile, it is difficult to make the best decision of the decision parameters. In the meantime, fuzzy related data assists in obtaining the best solution. All of this leads us to use fuzzy numbers to deal with ambiguous data (Nasseri and Ebrahimnejad 2010; Zadeh 1965).

In goal programming formulations, aspiration levels are regarded as precise, deterministic, and well known. However, in some decision-making situations, the parameters can be fuzzy, imprecise, or stochastic. In fact, there are many decision-making situations in which the DM lacks complete knowledge of some parameters, particularly the GP model’s goal values. In 1980, Narasimhan (1980) proposed a FGP formulation based on the concept of membership functions to address this issue.

### Mathematical Fuzzy Goal Programming

FGP is an extension of traditional goal programming that is used to solve decision problems with multiple objectives in an imprecise environment.

The fuzzy goal programming can be formulated as follows:

$$\begin{array}{l}\begin{array}{cc}Z_i(X)\gtrsim g_i,&\;,i=1,2,\dots K\end{array}\\\begin{array}{cr}\mathrm{Subject}\;\mathrm{to}&\end{array}\\\begin{array}{cc}&x\;\in X=\{x\in R^n;A_x\leq b;x\geq0\}\end{array}\end{array}$$

where $X$ is $\mathrm{an }n-$ dimensional decision vector. The symbol “ $\gtrsim$ ” ${ g}_{i}$ refers to the fuzzification of the aspiration level (i.e., approximately greater than or equal to).

For fuzzy-min, the membership function is defined as

$$\begin{array}{ccc}\mu\left(Z_i\left(X\right)\right)=&\left\{\begin{array}{c}1\\\frac{U_i-Z_i(x)}{U_i-g_i}\\0\end{array}\right.&\begin{array}{cc}\begin{array}{c}\mathrm{if}\;Z_i(x)\leq g_i\\\mathrm{if}\;g_i\leq Z_i(x)\leq U_i\\\mathrm{if}\;Z_i\left(x\right)\geq U_i\end{array}&i=K_0+1,2,\dots K_1\end{array}\end{array}$$

where ${U}_{i}$ is the upper tolerance limit.

For fuzzy-max, the membership function is defined as

$$\begin{array}{ccc}\mu\left(Z_i\left(X\right)\right)=&\left\{\begin{array}{c}1\\\frac{Z_i(x)-L_i}{g_i-L_i}\\0\end{array}\right.&\begin{array}{cc}\begin{array}{c}\mathrm{if}\;Z_i\left(x\right)\;\geq\;g_i\\\mathrm{if}\;L_i\;\leq\;Z_i\;(x)\;\leq\;g_i\\\mathrm{if}\;Z_i\;\left(x\right)\;\leq\;L_i\end{array}&i=1,2,\dots K_0\end{array}\end{array}$$

where ${L}_{i}$ is the lower tolerance limit for the *k*th fuzzy goal ${Z}_{i}\left(X\right)$.

Here, $tol$ is the tolerance interval. Using these definitions, the FGP model can be written as

$$\begin{array}{l}\mathrm F\mathrm i\mathrm n\mathrm d\;x\in X\\\mathrm S\mathrm o\;\mathrm a\mathrm s\;\mathrm t\mathrm o\;\mathrm M\mathrm a\mathrm x\mathrm i\mathrm m\mathrm i\mathrm z\mathrm e\;\mu\;\left(Z_i\left(X\right)\right)\\\begin{array}{l}\mathrm{Subject}\;\mathrm{to}\\\begin{array}{cc}\mu\left(Z_i\left(X\right)\right)\leq\frac{Z_i\left(x\right)-L_i}{g_i-L_i}&\mathrm{if}\;Z_i\left(x\right)\geq g_i\\\mu\left(Z_i\left(X\right)\right)\leq\frac{U_i-Z_i(x)}{U_i-g_i}&\mathrm{if}\;Z_i\;(x)\leq g_i\\\mu\left(Z_i\left(X\right)\right)\leq\frac{{(b}_i+tol\times b_i)-A_x}{tol\times b_i}&\mathrm{if}\;b_i\leq A_x\leq b_i+tol\times b_i\\x\in X=\{x\in R^n;&A_x\leq b;x\geq0\}\\\mu\left(Z_i\left(X\right)\right)\geq0&\end{array}\end{array}\end{array}$$

## Neutrosophic Goal Programming Technique

A minimizing type multi-objective linear programming is of the form

$$\begin{array}{c}\mathrm{min }\left[{Z}_{1}\left(x\right), {Z}_{2}\left(x\right), . . . , {Z}_{k}\left(x\right)\right]\\ \begin{array}{cc}{g}_{j}\left(x\right)\le {b}_{j} ,& j = \mathrm{1,2}, . . . , m\end{array}\end{array}$$

The concept of fuzzy decision $(D)$, fuzzy goal $(G)$, and fuzzy constraint $(C)$ was first discussed by Bellman and Zadeh (1970) and extensively used in many real-life decision-making problems under fuzziness. Therefore, a fuzzy decision set can be defined as follows:

$$D = G \cap C.$$

Equivalently, the neutrosophic decision set ${D}_{N}$, with the set of neutrosophic goals and constraints, can be defined as

$${D}_{N} = (\bigcap_{k=1}^{K}{G}_{k}) (\bigcap_{j=1}^{J}{C}_{j}) = (y, {T}_{D}\left(y\right), {I}_{D}\left(y\right), {F}_{D}\left(y\right)),$$

where

$$\begin{array}{c}\begin{array}{cc}{T}_{D}\left(y\right) =\mathrm{ min }\left\{\begin{array}{c}{T}_{{G}_{1}}\left(y\right), {T}_{{G}_{2}}\left(y\right),\dots , {T}_{{G}_{k}}\left(y\right)\\ {T}_{{C}_{1}}\left(y\right), {T}_{{C}_{2}}\left(y\right), {\dots , T}_{{C}_{j}}\left(y\right)\end{array}\right.& \forall y\in Y,\end{array}\\ \begin{array}{cc}{I}_{D}\left(y\right) =\mathrm{ min }\left\{\begin{array}{c}{I}_{{G}_{1}}\left(y\right), {I}_{{G}_{2}}\left(y\right),\dots , {I}_{{G}_{k}}\left(y\right)\\ {I}_{{C}_{1}}\left(y\right), {I}_{{C}_{2}}\left(y\right), {\dots , I}_{{C}_{j}}\left(y\right)\end{array}\right.& \forall y\in Y,\end{array}\\ \begin{array}{cc}{F}_{D}\left(y\right) =\mathrm{ min }\left\{\begin{array}{c}{F}_{{G}_{1}}\left(y\right), {F}_{{G}_{2}}\left(y\right),\dots , {F}_{{G}_{k}}\left(y\right)\\ {F}_{{C}_{1}}\left(y\right), {F}_{{C}_{2}}\left(y\right), {\dots , F}_{{C}_{j}}\left(y\right)\end{array}\right.& \forall y\in Y,\end{array}\end{array}$$

where ${T}_{D}\left(y\right)$, ${I}_{D}\left(y\right)$, and ${F}_{D}\left(y\right)$ are the truth, indeterminacy, and falsity membership functions of neutrosophic decision set ${D}_{N}$, respectively.

The bounds for each objective function were defined in order to formulate the various membership functions for multi-objective optimization problems. ${L}_{k}$ and ${U}_{k}$ represent the lower and upper bounds for each objective function, and can be calculated as follows: first, we solved each objective function as if it were a single objective within the problem’s constraints. We now have the $k$ solution set,${X}^{1}, {X}^{2}, \dots , {X}^{k}$, after solving $k$ objectives individually. The obtained solutions are then substituted for each objective function to provide the lower and upper bounds for each objective function, as shown below:

$$\begin{array}{c}\begin{array}{cc}{U}_{k}=\mathrm{max}\left[{Z}_{k}({X}^{k})\right]& \forall k=1, 2, \dots , K\end{array}\\ \begin{array}{cc}{l}_{k}=\mathrm{min}\left[{Z}_{k}({X}^{k})\right]& \forall k=1, 2, \dots , K\end{array}\end{array}$$

**Fig. 1:**
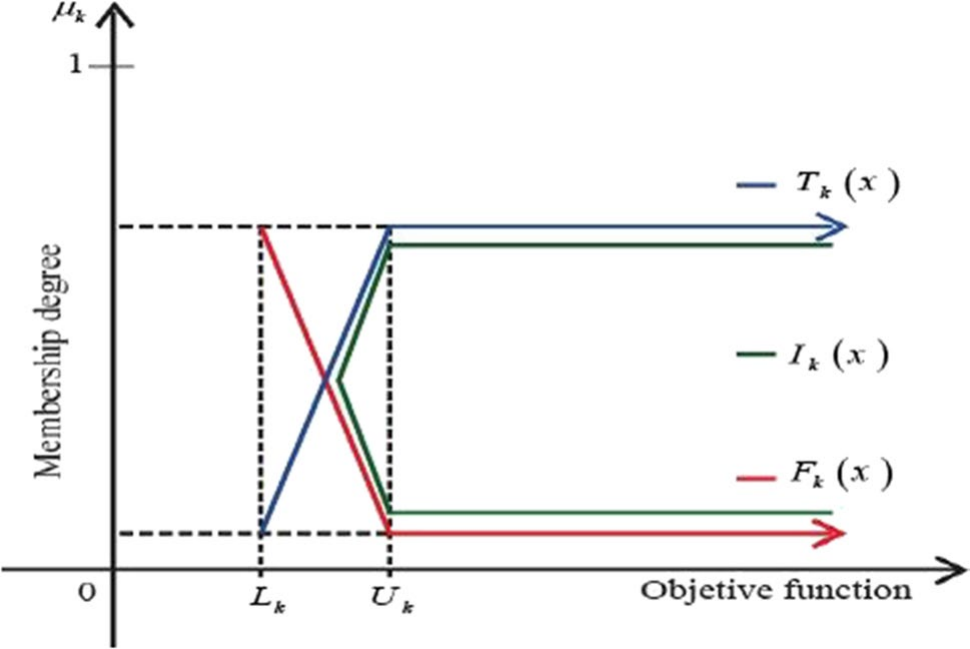
Diagrammatic representation of truth, indeterminacy, and falsity membership degree for the objective function

The bounds for $k$ objective functions under the neutrosophic environment can be obtained as follows:

$$\begin{array}{c}\begin{array}{ccc}{L}_{k}^{T} = {L}_{k},& {U}_{k}^{T} = {U}_{k}& \mathrm{for\;truth\;membership},\end{array}\\ \begin{array}{ccc}{L}_{k}^{I} = {L}_{k}^{T},& {U}_{k}^{I} = {L}_{k}^{T}+{s}_{k}& \mathrm{for\; indeterminacy\;membership},\end{array}\\ \begin{array}{ccc}{L}_{k}^{F} = {L}_{k}^{T}+ {t}_{k},& {U}_{k}^{F} = {U}_{k}^{T}& \mathrm{for\;falsity\;membership},\end{array}\end{array}$$

where ${s}_{k}$ and ${t}_{k}$ $\in (0, 1)$ are predetermined real numbers assigned by decision maker(s). By using the above lower and upper bounds, we defined the linear membership functions under a neutrosophic environment as shown in Fig. 1:

$$\begin{array}{l}T_k(Z_k(x))=\left\{\begin{array}{ll}1&if\;Z_k\left(x\right)<L_k^T\\\frac{U_k^T-Z_k(x)}{U_k^T-L_k^T}&if\;L_k^T\leq Z_k\left(x\right)\leq U_k^T\\0&if\;Z_k\left(x\right)>U_k^T\end{array}\right.\\I_k(Z_k(x))=\left\{\begin{array}{ll}1&\;if\;Z_k\left(x\right)<L_k^I\\\frac{U_k^I-Z_k(x)}{U_k^I-L_k^I}&if\;L_k^I\leq Z_k\left(x\right)\;\;\;\;\leq U_k^I\\0&if\;Z_k\left(x\right)>U_k^I\end{array}\right.\\F_k(Z_k(x))=\left\{\begin{array}{ll}1&if\;Z_k\left(x\right)<L_k^F\\\frac{U_k^F-Z_k(x)}{U_k^F-L_k^F}&if\;L_k^F\leq Z_k\left(x\right)\;\;\leq U_k^F\\0&if\;Z_k\left(x\right)>U_k^F\end{array}\right.\end{array}$$

In the above case, ${L}_{k}^{T}\ne {U}_{k}^{T}, {L}_{k}^{I}\ne {U}_{k}^{I},$ and ${L}_{k}^{F}\ne$ ${U}_{k}^{F}$ for all $k$ objective functions.

Figure 1 demonstrates a diagrammatic representation of the objective function with various components of membership functions in a neutrosophic environment. Furthermore, all three of the previously discussed membership degrees can be converted into membership goals based on their respective levels of attainment. The highest attainment degree of the truth membership function is unity (1), the indeterminacy membership function is neutral and independent with the highest attainment degree half (0.5), and the falsity membership function has the highest attainment degree zero (0). Now, in a neutrosophic environment, the transformed membership goals can be expressed as follow:

$$\begin{array}{c}{T}_{k}({Z}_{k}(x)) + {d}_{kT}^{-} - {d}_{kT}^{+} = 1,\\ {I}_{k}({Z}_{k}(x)) + {d}_{kI}^{-} - {d}_{kI}^{+} = 0.5,\\ {F}_{k}({Z}_{k}(x)) + {d}_{kF}^{-} - {d}_{kF}^{+} = 0,\end{array}$$

where ${d}_{kT}^{-}$,${d}_{kT}^{+}, {d}_{kI}^{-}$,${d}_{kI}^{+}$,${d}_{kF}^{-}$, and ${d}_{kF}^{+}$ are the over and under deviations such that ${d}_{kT}^{-}$. ${d}_{kT}^{+}$= 0, ${d}_{kI}^{-}$. ${d}_{kI}^{+}$ = 0, and ${d}_{kF}^{-}$. ${d}_{kF}^{+}$ = 0, for truth membership, indeterminacy membership, and falsity membership goals under a neutrosophic environment.

The general formulation of the NGP model for multi-objective optimization problem (1) is represented as follows:

$$\begin{array}{c}\mathrm{Minimize }Z = \sum_{k=1}^{K}{w}_{kT }. {d}_{kT}^{-} + \sum_{k=1}^{K}{w}_{kI }. {d}_{kI}^{-} + \sum_{k=1}^{K}{w}_{kF }. {d}_{kF}^{+}\\ \mathrm{Subject to}\\ \begin{array}{c}{T}_{k}({Z}_{k}(x)) + {d}_{kT}^{-} - {d}_{kT}^{+} \ge 1\\ {I}_{k}({Z}_{k}(x)) + {d}_{kI}^{-} - {d}_{kI}^{+} \ge 0.5\\ \begin{array}{c}{F}_{k}({Z}_{k}(x)) + {d}_{kF}^{-} - {d}_{kF}^{+} \le 0\\ {T}_{k}({Z}_{k}(x)) \ge {I}_{k}({Z}_{k}(x))\\ \begin{array}{c}{T}_{k}({Z}_{k}(x)) \ge {F}_{k}({Z}_{k}(x))\\ {F}_{k}({Z}_{k}(x)) \ge 0\\ \begin{array}{c}{T}_{k}({Z}_{k}(x)) + {I}_{k}({Z}_{k}(x)) + {F}_{k}({Z}_{k}(x)) \le 3\\ {d}_{kT}^{-} . {d}_{kT }^{+}= 0\\ \begin{array}{c}{d}_{kI}^{-} . {d}_{kI}^{+} = 0\\ {d}_{kF}^{-} . {d}_{kF}^{+}= 0\\ \begin{array}{c}\begin{array}{cc}{g}_{j}(x) \le {b}_{j},& j = 1, 2, \dots , {m}_{1}\end{array}\\ \begin{array}{cc}{g}_{j}(x) \ge {b}_{j} ,& j = {m}_{1} +1, {m}_{1} + 2, \dots , {m}_{2}\end{array}\\ \begin{array}{c}\begin{array}{cc}{ g}_{j}(x) = {b}_{j} ,& j = {m}_{2} + 1, {m}_{2} + 2, \dots ,\mathrm{ m}\end{array}\\ {x}_{i} \ge 0, i = 1, 2, \dots , q, {x}_{i}\in X\\ {d}_{kT}^{-}, {d}_{kT }^{+}, {d}_{kI}^{-}, {d}_{kI}^{+} , {d}_{kF}^{-}, {d}_{kF}^{+} \ge 0, \forall k,\end{array}\end{array}\end{array}\end{array}\end{array}\end{array}\end{array}\end{array}$$

where ${w}_{kT}$, ${w}_{kI}$, and ${w}_{kF}$ are the weights assigned to deviations of the truth, indeterminacy, and falsity membership goals of each objective function, respectively. Now the assignment of corresponding weighting schemes of different weights can be obtained as follows:

$$\begin{array}{ccc}{w}_{kT} = \frac{1}{{U}_{k}^{T}-{L}_{k}^{T}} ,& {w}_{kI} = \frac{1}{{U}_{k}^{I}-{L}_{k}^{I}},& {w}_{kF} = \frac{1}{{U}_{k}^{F}-{L}_{k}^{F}}\end{array}$$

Hence, the optimum evaluation of multi-objective optimization problems by using NGP approach is a very useful technique as it involves the degree of indeterminacy, which is independent and certainly ensures the achievement of marginal evaluation of each membership goal by reducing the deviational values under a neutrosophic environment.

## Egypt’s Sustainable Development Goals: a Case Study

Egypt has achieved the majority of the Millennium Development Goals, a set of eight global goals that ran from 2000 to 2015 and ranged from country requirements to halve extreme poverty to reducing HIV/AIDS spread and achieving universal primary education. According to the Millennium Development Goals, Egypt reduced extreme poverty (people living on less than $25.1 per day) by more than 62 percent between 1990 and 2008, meeting targets within the framework of the Millennium Development Goals’ first goal. Egypt had a net primary enrollment rate of 97 percent in 2010. Between 1990 and 2013, the under-five mortality rate fell by more than 74%. The mortality rate from tuberculosis has dropped by more than 81 percent. More than 95 percent of the population now has access to clean water and sanitation. Some targets, however, remained unmet, particularly those related to MDG 3, “Promote gender equality and empower women.” The gender ratio in primary education has converged, but it has not yet reached the target of gender parity by 2013. During the same period, the proportion of women working in the non-agricultural sector fell by 9%, falling to around 19% in 2013.^7^ Egypt’s population is expected to increase by nearly 24% by 2030, from 93.8 million in 2015 to 122.6 million. With this expansion, the country will retain some of its youth. In 2030, 30% of the population will be under the age of 15, and more than 60% will be of working age (from 15 to 64). Egypt’s economy is expected to grow at a rate of 5 to 6 percent per year over the forecast period, with a gross domestic product of $571 billion by 2030. GDP increased from $10,250 in 2015 to $14,270 by 2030, roughly equal to Brazil’s level in 2018.

This growth is forecast to lead to a reduction in poverty and an expansion of the middle class. Ten million fewer people will be living in poverty (as defined by the population living on Egypt’s current national poverty line, equivalent to less than $3.40 per day in 2011 US dollars) in 2030 compared with 2015, while the middle-class population (those living on between $10 and $50 per day) is forecast to more than double by 2030.

Egypt may also face economic challenges such as the informal economy, unemployment, and low female labor-force participation. Informal employment as a percentage of the non-agricultural labor force is expected to fall from 47 percent today to 36 percent by 2030. In the informal sector, an additional 2.5 million people are expected to work. Women’s labor-force participation, which currently stands at around 20%, is not expected to increase significantly.

Human development, in terms of the health and education of the population, is projected to improve steadily. Educational attainment, as measured by the average Egyptian’s years of schooling, increases from 7.1 years in 2015 (6.5 for women and 7.8 for men) to 8.5 years in 2030 (8 for women and 9 for men). Thus, even while educational attainment increases across the board, female attainment continues to lag behind. Life expectancy increases from 71.3 years to 74 by 2030. And the under-5 mortality rate falls from 22 deaths per 1000 live births in 2015 to 14.7 by 2030.

## Data Collection and Model Formulation

We looked at employment as a decision variable, which is critical for long-term development. The following goals for gross domestic product, electricity consumption, and greenhouse gas emissions are among the primary goals for achieving the Sustainable Development Goals, which are required for the modern economy to function properly.

### Data Collection

#### Gross Domestic Product

The Ministry of Planning and Economic Development – National Accounts Data and the IMF publish GDP by sector. In some cases, the most recent entry was unavailable, so we used the estimated annual percentage growth rate of GDP in local currency based on constant growth. Table 1 shows GDP per capita estimates by sector for the year 2019.

**Table 1:** Sectoral contribution of economic sectors to the identified goals

Decision variable	Economic sectors	GDP (in million EGP)	EC (in GWh)	GHG emissions (MT ${\mathrm{CO}}_{2}$ e)	Number of employment (in thousand)
${x}_{1}$	Agriculture, forestry, and fishing	669,783.5	542.0	30.6	5542.5
${x}_{2}$	Manufacturing	942,408.0	5871.0	*	3431.3
${x}_{3}$	Construction	371,457.5	9873.0	**	3310.5
${x}_{4}$	Transport, storage	277,865.2	46.0	53.9	2397.1

GHG emissions in manufacturing (*) and construction (**) together is 41.12

#### Electricity Consumption

Table 1 summarizes the per-capita estimates for electricity consumption across sectors in gigawatt hours (GWh). The Ministry of Electricity and Renewable Energy provided sectoral data for electricity consumption for the year 2019.^8^

#### Greenhouse Gas Emissions

GHG emission data for 2018 were obtained from the United Nations Framework Convention on Climate Change (UNFCCC),^9^,^10^ Climate Watch,^11^ and CAIT. Table 1 summarizes per-capita GHG emissions by sector in gigagrams of ${\mathrm{CO}}_{2}$ equivalent. We used the conclusion shown in the paper presented by Lamia Abdullah (2020),^12^ which states “reducing the overall emissions by 20% by 2030” to estimate the value of greenhouse gases in 2030 due to the lack of an officially measured target that can be relied upon.

#### Number of Employees

The number of employees across the economic sectors were obtained from International Labour Organization,^13^ Central Agency for Public Mobilization and Statistics,^14^ and UNDP. Table 1 presents the number of employees (in thousands) employed in each sector. The annual growth percentage of labor was used to project the data with reference to year 2019. We have used the following law to find the number of employees in 2030 because there is no specific value in any source.

$$\mathrm{Number\;of\;employees\;}=\mathrm{\;labor\;force\;}\times (1\;-\mathrm{\;unemployment\;rate})$$

**Fig. 2:**
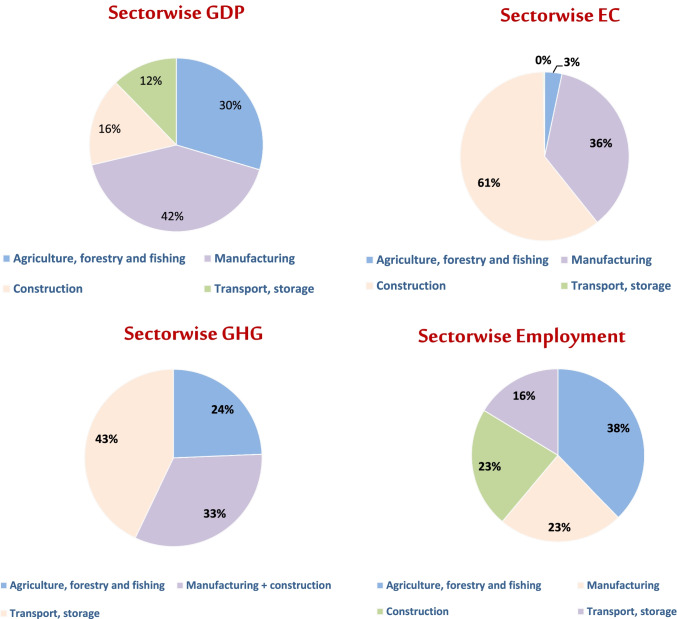
Percentage contribution of the four chosen sectors

Table 2 shows the projected 2030 goal values as well as the corresponding growth rates for the four criteria. GDP growth rates were calculated using data from Egypt Vision 2030.^15^ The Energy Strategy 2035 was also used to estimate the growth rates of electricity consumption in each of the four sectors. The number of employees was estimated using CAPMS and UNDP, and the amount of GHG emissions was estimated using the research paper introduced by Lamia Abdullah (2020) and the UNFCCC. In addition, we used the following rule to calculate the value of the compound annual growth rate formula. In Fig. 2, we show the sector-wise contribution of the GDP, EC, GHG emissions, and the number of employees.

**Table 2:** Identified goals for $\mathrm{SDG}$ s (2030)

Goals	Value	Goals by 2030	Compound annual growth rate
GDP (in million EGP)	5,526,954.7	8,132,519.0	0.0357
EC (in GWh)	16,332.0	27,123.0	0.0472
GHG emissions (MT ${\mathrm{CO}}_{2}$ e)	125.6	200.0	0.0395
Number of employment (in thousand)	14,681.4	36,080.0	0.0852

$$\mathrm{Compound\;annual\;growth\;rate\;}= {[\mathrm{ending\;value}/\mathrm{\;beginning\;value}]}^{\frac{1}{\mathrm{number\;of\;years}}} - 1.$$

### Model Formulation and Numerical Results

In our model, we use a formulation as a multi-objective integer linear programming model to determine the optimal allocation of employees across different economic activities in order to maintain GDP growth, electricity consumption, and GHG emissions. The objectives are as follows:

$$\begin{array}{l}Z_1(X)=\sum\nolimits_{j=1}^4(\frac{{(\mathrm{GDP})}_j}{e_j})x_j,\\Z_2(X)=\sum\nolimits_{j=1}^4(\frac{{(\mathrm{EC})}_j}{e_j})x_j,\\Z_3(X)=\sum\nolimits_{j=1}^4(\frac{{(\mathrm{GHG})}_j}{e_j})x_j,\\\mathrm{Subject}\;\mathrm{to}:\;\\\begin{array}{cc}&\begin{array}{l}\sum\nolimits_{j=1}^4x_j\leq e_G\\\sum\nolimits_{j=1}^4{(\mathrm{GDP})}_jx_j\leq{\;\;\;(\mathrm{GDP})}_G\\{e_J\leq x}_j\leq e_{GJ},\;\;\;\;\forall j=1,2,3,4\end{array}\end{array}\end{array}$$

Here, the following symbols are used:

- Objective function ${Z}_{1}$ optimizes the per-capita gross domestic product across the *j*th economic sector.
- Objective function ${Z}_{2}$ optimizes the per-capita gross electricity consumption across the *j*th economic sector.
- Objective function ${Z}_{3}$ optimizes the per-capita greenhouse gas emissions across the *j*th economic sector.
- ${x}_{j}$ is the number of employees in the *j*th contributing sectors.
- ${e}_{j}$ is the current employment in the *j*th sector.
- ${xe}_{Gj}$ is the employment goal in the *j*th sector.
- ${(\mathrm{GDP})}_{j}$ is the gross domestic product in the *j*th sector.
- ${(\mathrm{EC})}_{j}$ is the electricity consumption in the *j*th sector.
- ${(\mathrm{GHG})}_{j}$ is the GHG emissions in the *j*th sector.
- ${(\mathrm{GDP})}_{j}$ is the GDP goal for sustainable development.

The mathematical formulation for the model is as follows:

$$\begin{array}{l}Z_1(X)=120.8450158x_1+274.650424x_2+112.2058601x_3+115.9172333x_4\geq8,150,140.0\\{\mathrm Z}_2(X)=0.09778981x_1+1.71101332x_2+2.98232865x_3+0.01918985x_4\geq39000.0\\{\mathrm Z}_3(X)=5.5173658\times10^{-3}x_1+6.099261325\times10^{-3}(x_2+x_3)+0.0224771599x_4\geq145.41\\\mathrm{Subject}\;\mathrm{to}:\\\begin{array}{lc}&x_1+x_2+x_3+x_4\leq36,080.0\end{array}\\\begin{array}{lc}&669,783.5x_1+942,408.0x_2+371,457.5x_3+277,865.2x_4\leq18,954,400.0\end{array}\\\mathrm{and}\;\mathrm{the}\;\mathrm{bounds}:\\\begin{array}{ccc}&x_1\geq5542.5,&x_2\geq3431.3\end{array}\\\begin{array}{ccc}&x_3\geq3310.5,&x_4\geq2397.1\end{array}\end{array}\\$$

#### The Fuzzy Goal Programming Approach for the Model

Firstly, calculate the lower bound and upper tolerance limit of goals from the previous goal programming model. Table 3 shows the lower bound and upper tolerance limit of goals.

**Table 3:** Lower and upper tolerance limit of goals

	Lower bound	Upper bound
${Z}_{1}$	2,261,514.0	8,138,649.0
${Z}_{2}$	16,332.0	80,149.7
${Z}_{3}$	125.6	606.6

Secondly, formulate the fuzzy goal programming approach using ($tol$ = 0.1).

$$\begin{array}{l}\mathrm{Max}\lambda\\\mathrm{Subjectto}\\\begin{array}{cc}&\begin{array}{l}5,888,626.0\lambda\leq Z_1-2,261,514.0\\-11,491.0\lambda\leq8,138,649.0-Z_1\\22,668.0\lambda\leq Z_2-16,332.0\\41,149.66\lambda\leq80,149.66-Z_2\\19.83\lambda\leq{\mathrm Z}_3-125.5800\\461.1498\lambda\leq606.5598-Z_3\\3608.0\lambda\leq39,688.0-(x_1+x_2+x_3+x_4)\\189,5440.0\lambda\leq20,849,840.0-(669,783.5x_1+942,408.0x_2+\\371,457.5x_3+277,865.2x_4)\\554.25\lambda\leq x_1-6096.75\\343.13\lambda\leq x_2-3774.43\\331.05\lambda\leq x_3-3641.55\\239.71\lambda\leq x_4-2636.81\end{array}\end{array}\end{array}$$

#### The NGP Approach for the Model

$$\begin{array}{l}\mathrm{Minimize\;}Z = {W}_{IT}\times\;{d}_{1T}^{-} + {W}_{2T}\times {d}_{2T}^{-} + {W}_{3T}{d}_{3T}^{-} + {W}_{II}\times\;{d}_{1I}^{-}+ {W}_{2I}\times\;{d}_{2I}^{-} + {W}_{3I}\times\;{d}_{3I}^{-} + {W}_{IF}{d}_{1F}^{-} + {W}_{2F}\times\;{d}_{2F}^{-} + {W}_{3F}{ d}_{3F}^{-}\\ \mathrm{Subject\;to}\\ \begin{array}{c}\mathrm{8,138,649\;}- {Z}_{1} +\mathrm{\;5,877,135\;}{d}_{1T}^{-} -\mathrm{ 5,877,135\;}{d}_{1T}^{\mp\;} \ge\;\mathrm{\;5,877,135}\\ \mathrm{2,261,514.2\;}- {Z}_{1} + 0.2 {d}_{1I}^{-} - 0.2\;{d}_{1I}^{+}\;\ge 0.1\\ \begin{array}{c}{Z}_{1} -\mathrm{\;2,261,514.1\;}+\mathrm{\;5,877,134.9\;}{d}_{1F}^{-} -\mathrm{\;5,877,134.9\;}{d}_{1F}^{\mp\;}\;\le 0\\ \mathrm{80,149.66\;}- {Z}_{2} +\mathrm{\;63,817.66\;}{d}_{2T}^{-} -\mathrm{\;63,817.66\;}{d}_{2T}^{+}\;\ge \mathrm{ 63,817.66}\\ \begin{array}{c}\mathrm{16,332.4 }- {Z}_{2} + 0.4 {d}_{2I}^{-} - 0.4 {d}_{2I}^{\mp } \ge 0.2\\ {Z}_{2} -\mathrm{ 16,332.5 }+\mathrm{ 63,817.16 }{d}_{2F}^{-} -\mathrm{\;63,817.16 }{d}_{2F}^{+} \le\;0\\ \begin{array}{c}606.5598\;- {Z}_{3 }+ 480.9798\;{d}_{3T}^{-} - 480.9798\;{d}_{3T}^{\mp\;}\;\ge 480.9798\\ 125.68- {Z}_{3} + 0.1 {d}_{3I}^{-} - 0.1 {d}_{3I}^{+} \ge 0.05\\ \begin{array}{c}{Z}_{3} -\;125.78 + 480.7798 {d}_{3F}^{-} - 480.7798 {d}_{3F}^{-} \le 0\\ (\mathrm{8,138,649 }- {Z}_{1})/\mathrm{5,877},135 \ge\;(\mathrm{2,261,514.2 }- {Z}_{1})/0.2\\ \begin{array}{c}(\mathrm{8,138,649\;}- {Z}_{1})/\mathrm{5,877,135 }\ge\;({Z}_{1} -\mathrm{\;2,261,514.1})/\mathrm{5,877,134.9}\\ ({Z}_{1} -\mathrm{ 2,261,514.1})/\mathrm{5,877,134.9 }\ge 0\\ \begin{array}{c}(\mathrm{80,149.66 }- {Z}_{2})/\mathrm{63,817.66 }\ge (\mathrm{16,332.4}- {Z}_{2})/0.4\\ (\mathrm{80,149.66\;}- {Z}_{2})/\mathrm{63,817.66 }\ge\;({Z}_{2} -\mathrm{ 16,332.5})/\mathrm{63,817.16}\\ \begin{array}{c}({Z}_{2} -\mathrm{\;16,332.5})/\mathrm{63,817.16 }\ge 0\\ (606.5598 - {Z}_{3})/480.9798\;\ge (125.68 - {Z}_{3}/0.1\\ \begin{array}{c}(606.5598\;-\;{Z}_{3})/480.9798 \ge\;({Z}_{3} - 125.78)/480.7798\\ ({Z}_{3} - 125.78)/480.7798 \ge 0\\ \begin{array}{c}(\mathrm{8,138,649 }- {Z}_{1})/\mathrm{5,877,135 }+ (\mathrm{2,261,514.2 }- {Z}_{1} )/0.2 + ({Z}_{1} -\mathrm{ 2,261,514.1})/\mathrm{5,877,134.9 }\le 3\\ (\mathrm{80,149.66 }- {Z}_{2})/\mathrm{63,817.66 }+ (\mathrm{16,332.4 }- {Z}_{2})/0.4 + ({Z}_{2} -\mathrm{ 16,332.5})/\mathrm{63,817.16 }\le 3\\ \begin{array}{c}(606.5598 - {Z}_{3})/480.9798 + (125.68 - {Z}_{3})/0.1 + ({Z}_{3} - 125.78)/480.7798 \le 3\\ {x}_{1}+ {x}_{2} + {x}_{3} + {x}_{4} \le \mathrm{ 36,080.0}\\ \begin{array}{c}\mathrm{669,783.5}{ x}_{1 }+\mathrm{ 942,408.0 }{x}_{2} +\mathrm{ 371,457.5 }{x}_{3} +\mathrm{ 277,865.2 }{x}_{4} \le \mathrm{ 18,954,400.0}\\ {x}_{1}\ge 5542.5\\ \begin{array}{c}{x}_{2}\ge 3431.3\\ {x}_{3} \ge 3310.5\\ \begin{array}{c}{x}_{4} \ge 2397.1\\ \begin{array}{c}{d}_{kT}^{-} \times\;{d}_{kT}^{+} = 0\\ {d}_{kI}^{-} \times {d}_{kI}^{+} = 0\\ \begin{array}{c}{d}_{kF}^{-}\;\times\;{d}_{kF}^{+} = 0\\ {d}_{kT}^{-}, {d}_{kT}^{+}, {d}_{kI}^{-}, {d}_{kI}^{+}, {d}_{kF}^{-}, {d}_{kF}^{+} \ge 0 \;\forall\;K\end{array}\end{array}\end{array}\end{array}\end{array}\end{array}\end{array}\end{array}\end{array}\end{array}\end{array}\end{array}\end{array}\end{array}\end{array}\end{array}\end{array}$$

## Results and Discussion

In this section, we apply FGP and NGP approaches presented in the “Neutrosophic Goal Programming Technique” and “Egypt’s Sustainable Development Goals: a Case Study” sections to the above model formulation. The resulting optimization problem is solved using the numerical optimization software LINGO 16.0 (Lingo System Inc. Lingo-User’s Guide 2016).

As it can be seen in Tables 4 and 5, NGP approach is more accurate than FGP approach. This model suggests that the achievement of the goal set for economic growth until year 2030 will not be possible without any additional measures in every economic sector of the country.

**Table 4:** A comparison of employment in different sectors between FGP and NGP approaches

	${X}_{1}$	${X}_{2}$	${X}_{3}$	${X}_{4}$
FGP approach (6.2.1)	6534.49	19,505.11	3903.01	2826.13
NGP approach (6.2.2)	5542.50	3431.30	3310.61	2405.97

**Table 5:** A comparison between objective values of FGP and NGP approaches

	${Z}_{1}$	${Z}_{2}$	${Z}_{3}$
FGP approach (5.2.1)	6,912,285.16	45,706.80	242.35
NGP approach (5.2.2)	2,262,554.61	16,332.50	125.78

### Gross Domestic Product Growth

The above model indicates that achieving the targets set for GDP growth by 2030 is not possible without some extraordinary efforts towards GDP growth. These efforts may include capital formation, technological improvement, expanding the establishment of industrial zones in new cities, working to amend some regulations and laws regulating industrial activities, and searching for procedures related to encouraging and integrating the industrial activities of medium and small enterprises into the formal economy. This can include activating granting incentives and exemptions for small, medium, and micro enterprises, facilitating the provision of raw materials for industry, activating import control tools, and studying the localization of technical schools in major industrial complexes. Creating digital economy is essential to help realize the envisioned economic future.

### Electricity Consumption

It is clear from the model the need for extraordinary policy measures towards the EC to fulfill the incredibly high demand. As a result, the model proposes that the goal of meeting energy consumption by 2030 will not be met unless additional efforts are made to diversify electricity generation sources. Alternative and renewable energy sources are critical to meeting rising demand. This is consistent with the current emphasis and efforts on additional investments in clean and renewable energy sources to address growing energy concerns. Furthermore, the poor quality of the power supply, frequent interruptions, and lack of electricity place a significant burden on the rapidly expanding trade and industry. As a result, effective policies that assist the state in producing electricity may be required.

### GHG Emissions

The model shows that reducing GHG emissions by 2030 would require additional measures in each sector. To meet the 2030 target for GHG, rely on renewable energy sources, establish strict emission standards, implement a national energy conservation program, and build smart cities. The model’s policy-specific message is a focus on diversifying energy portfolios by adding alternate energy sources, as well as a strong push to reduce domestic GHG emissions. Finally, it can be stated that achieving any of the three objectives is difficult unless and only if the number of employees in each sector of economic activity is optimal.

## Conclusions

In this paper, we presented a novel neutrosophic goal programming model that incorporates optimal resource allocation to simultaneously satisfy prospective goals on economic development, energy consumption, workforce, and GHG emission reduction by 2030 by application to the main economic sectors in Egypt. We also compared the results of FGP and NGP approaches. We demonstrated that neutrosophic goal programming approach is more accurate than fuzzy goal programming approach because it deals with incomplete and indeterminate information and has three independent degrees: truth membership degree, indeterminacy-membership degree, and falsity-membership degree.

According to the model, Egypt should take the necessary steps toward renewable technologies, specifically solar and wind energy, which have enormous potential for achieving sustainability goals. This can also help with meeting the greenhouse gas emissions target, as well as meeting the electricity consumption target. The model also provides a quantitative and mathematical justification for additional investments to change Egypt’s energy portfolio. The model’s analysis emphasizes the importance of further research into alternative (green) energy sources.

## Data Availability

All data generated or analyzed during this study are included in this published article.

## Footnotes

1 https://www.capmas.gov.eg/

2 https://www.worldbank.org/en/country/egypt/publication/economic–update–october–2019.

3 http://www.moee.gov.eg/test_new/home.aspx

4 https://mped.gov.eg/

5 https://www.idsc.gov.eg/

6 https://web.archive.org/web/20190619035125/https://www.ceicdata.com/en/indicator/egypt/unemployment–rate.

7 https://www.eg.undp.org/content/egypt/en/home/library/sustainable-development-goals--sdgs-/sustainable-development-goals-report--egypt-2030.html

8 http://www.moee.gov.eg/english_new/home.aspx

9 https://unfccc.int/sites/default/files/resource/BUR%20Egypt%20%20AR.pdf

10 http://cait.wri.org/historical/Country%20GHG%20Emissions

11 https://www.climatewatchdata.org/countries/EGY

12 https://journals.ekb.eg/article_73503.html

13 https://www.ilo.org/shinyapps/bulkexplorer20/?lang=en&segment=indicator&id=EMP_2EMP_SEX_ECO_NB_A

14 https://www.capmas.gov.eg/Pages/Publications.aspx?page_id=5104&Year=23620

15 https://arabdevelopmentportal.com/sites/default/files/publication/sds_egypt_vision_2030.pdf
